# Sports participation of children and adolescents in Germany: disentangling the influence of parental socioeconomic status

**DOI:** 10.1186/s12889-021-11284-9

**Published:** 2021-07-23

**Authors:** Lea Rittsteiger, Thomas Hinz, Doris Oriwol, Hagen Wäsche, Claudia Santos-Hövener, Alexander Woll

**Affiliations:** 1 Karlsruhe Institute of Technology (KIT), Institute of Sports and Sports Science (IfSS), Engler-Bunte-Ring 15, 76131 Karlsruhe, Germany; 2 Department for History and Sociology, University of Konstanz, Konstanz, Germany; 3 Robert-Koch Institute (RKI), Berlin, Germany

**Keywords:** Children, Physical activity, Sports, Club sports, Socioeconomic status, Gender, Migration background

## Abstract

**Background:**

Participation in sports and physical activity (PA) is a critical resource for children’s health and social development. This study analyzes how the parental socioeconomic status (SES) of children and adolescents affects their PA in sports clubs (organized sports) and outside of sports clubs (unorganized sports) and tests whether the potential impact of parental SES is mediated by the opportunity structure of their residential area (walkability, infrastructure, etc.) and by family and peer support for PA. Furthermore, PA is analyzed respecting differences by gender and migration background.

**Methods:**

Using representative data from the MoMo/KiGGS study (2009–2012 and 2014–2017), we take into account about 8000 measurements from about 7000 subjects. We estimate hurdle regression models to analyze the minutes per week spent on sports activities.

**Results:**

Results show that children with a higher parental SES, children living in areas with many opportunities for PA, and children receiving family and peer support are more physically active than children without these features. Controlled for opportunities and support, status effects are small but visible. The differences regarding parental SES are much more apparent for organized sports than for unorganized sports, indicating the relevance of economic resources. Boys are more active than girls, whereas there is no clear effect of migration background.

**Conclusions:**

The coefficient of parental SES on organized sports most probably relates to the resources needed to participate in sports clubs, including fees and equipment. Lower membership fees might potentially help to integrate children with low parental SES into sports clubs and thereby make organized sports more accessible to all social classes.

## Background

Engaging in sports is among the dominant forms of physical activity (PA) in modern societies. While PA positively affects health *at every age* [1–4], an active lifestyle in *childhood* is specifically relevant. PA prevents many physical and mental health problems in the young [2, 5, 6] and has long-term positive health outcomes in adulthood [7–10]. Therefore, the World Health Organization (WHO) encourages regular PA, especially in children [11]. Furthermore, the personal and social development of children and adolescents also benefits from PA [12, 13]. Finally, participation in sports fosters social integration as well as the development of social skills and teamwork [14].

Opportunities for sports and PA are thus beneficial social resources for all children and adolescents. However, there is some evidence that their participation in sports is influenced by distinct socioeconomic inequalities [15–17]. For obvious reasons, it is critical to know which social mechanisms enable or prevent the participation of children and adolescents in sports and PA, specifically the analysis will point out how social factors such as parental socioeconomic status (SES), opportunity structure and parental support influence children’s and adolescents’ PA and sports participation.^1^ Achieved SES drives decisions of parents where to live (choice of residential neighborhood with more or less sports friendly opportunities) and how to support the development of their children (by encouraging them to be physically active). Thus, the analysis allows to identify direct and indirect effects of parental SES. Reliable information on influential factors is the prerequisite for informing health promotion and for fostering potential benefits regarding education and social integration through sports.

## Determinants of children’s PA in sports

To understand children’s PA behavior, various elements must be taken into account. Individual PA is a function of individual, social, and environmental factors [18, 19]. Evidently, one important aspect of children’s social and physical environment is their parents and their socioeconomic status (SES), which characterizes the level of *resources* available to them and their family’s *lifestyle*. Various studies have shown that children from families with a higher SES are more physically active in sports than children from families with a lower SES [15, 17, 20, 21]. This mainly applies to organized sports in sports clubs, but also to unorganized sports in leisure time outside of sports clubs [22]. A recent study by Andersen and Bakken [17] reveals that there are distinct class-specific patterns for youth’s participation in organized sports in Norway. A higher social class background is associated with a higher involvement in organized sports.

On the one hand, these results may be explained by the better equipment and resources available to parents with a higher SES [17] who invest in their children’s education, including in their PA. For instance, parents with more financial resources can more easily afford membership fees for sports clubs or expensive sports gear for their children. On the other hand, some explanations focus on differences in values, beliefs, and attitudes between people of differing SES [15]. The importance of health and the awareness of the health benefits of PA (health literacy) are considered to be more prevalent among the higher social classes [23]. Children from a higher social background therefore assign a higher importance to sports than children from a lower social background. Furthermore, parents with a higher SES are more likely to support their children’s PA in sports, for example by taking them to sports events, encouraging club membership, and acting as *role models* when they engage in their own physical activities.^2^ Children whose families and peers support PA in sports and do sports themselves are more physically active [26–29]. Beyond financial resources and values, families with a higher SES usually live in wealthy residential areas that provide their residents with more sports facilities, such as sports grounds, playgrounds, and gyms. It has been shown that the availability of opportunities for PA and sports in a residential area also has a positive effect on children’s sports and activity engagement [30, 31]. A missing link in previous research is a comprehensive investigation of socioeconomic variables that are correlated to each other – particularly parental financial resources, life style and support, and place of residence.

**Fig. 1:**
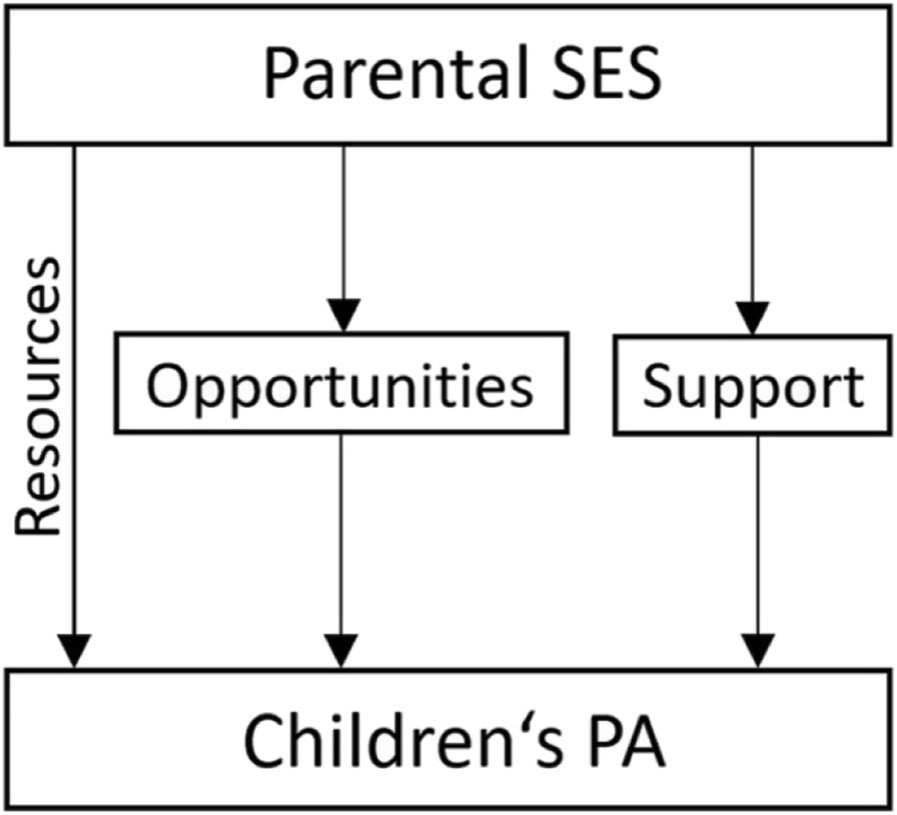
Conceptualization of the different effects of parental SES on children’s PA

Thus, this study contributes to the research through a more fine-grained analysis of the relations between parental SES and children’s sports participation, with mediating factors of the opportunity structures of the residential area and family and peer support for PA. Our main research question is to what extend the three components of parental SES (resources, residential area, family and peer support) influence children’s PA in organized and unorganized sports. Figure 1 depicts the assumed relations of the different aspects of SES and its influence on children’s PA engagement in sports. First, we propose that parental SES has a direct effect on children’s PA via resources. Children with a higher parental SES are more physically active in organized and unorganized sports than children with a lower parental SES (*H1*). Second, opportunities for PA in the residential area and family and peer support for PA are mediating factors that partly explain the influence of SES on PA. We expect that children living in an area with lots of opportunities for PA (*H2*), and children whose parents, siblings, and peers support PA (*H3*) are more physically active in organized and unorganized sports. Thus, the study provides new insights into how these three variables correlate and might impact the PA of children and adolescents.

In accordance with previous research on group characteristics, we also include children’s genders and migration backgrounds. A large number of studies have reported differences in sports engagement between male and female children: considering all sports activities, boys are more physically active in sports than girls [2, 32–34]. Children are socialized with gender role attitudes; they learn from their parents and their social environment [35]. This includes their attitude towards sports and their PA in sports [36]. Traditionally, different activities are considered appropriate for females and males, and sports are a primarily male domain [37]. In line with this, we propose that girls are less physically active in organized and unorganized sports than boys (H4).

Previous research has also shown that children with a migration background are less physically active in sports than children without a migration background [16, 38, 39]. However, studies differentiating between organized sports in a sports club and unorganized sports outside of a sports club found this difference for organized sports [40, 41] only. These results have been explained by differences in knowledge between children (and parents) with and without a migration background. Knowledge about the availability of sports clubs in the residential area and about formal processes such as membership application is necessary for organized PA in sports clubs. This knowledge can be more difficult to acquire for children (and parents) with a migration background, mainly because of language difficulties [42]. We therefore assume that children with a migration background are less physically active in *organized* sports than children without a migration background (*H5*), independent of their families’ financial resources. No difference is expected between children with and without a migration background regarding unorganized sports, as many types of unorganized sports can be done without overcoming formal hurdles (for example, running or biking).

We also focus on the interaction of gender and migration background. Girls with a migration background are supposed to be less physically active in sports than girls without a migration background; in contrast, results regarding differences by migration background are mixed for boys [43, 44]. Accordingly, we expect that girls with a migration background are less physically active in organized and unorganized sports than girls without a migration background, while we expect no difference for boys by migration background (*H6*).

Following our theoretical setup, the study at hand investigates the total effect size of parental SES on the PA of children in a multivariate model, while also taking into account opportunities for PA in the residential area, parental and peer support for PA, gender, and migration background. We add some further controls: district size, age, and birth cohort of children. The dependent variables are the minutes spent by the children in PA in organized and unorganized sports per week. Although we rely on adequate statistical techniques to control for observed heterogeneity of respondents, we are fully aware that the estimated “effect” coefficients represent a correlation structure and not causal mechanisms.

## Data and methods

We use data from the Motorik-Modul study (MoMo), a subsample of the German Health Interview and Examination Survey for Children and Adolescents (KiGGS). The study is representative for German children and adolescents [45] and combines a cohort design with a longitudinal panel design. So far, three waves were conducted (baseline 2003–2006, wave 1, 2009-2012, and wave 2, 2014-2017), whereby in each wave, panelists were interviewed repeatedly and new cohorts were added. The sampling procedure consisted of three steps. First, 167 sample units, stratified by their grade of urbanization (BIK classification) and their geographic distribution, were drawn from a register of German communities. Second, in each community, addresses were randomly selected from population directories [46]. This procedure formed the overall sample for the KiGGS study, from which respondents were randomly selected for participation in the MoMo study. To measure the PA of children and adolescents the MoMo-AFB questionnaire was used, covering everyday physical activity, sports within and outside of organized clubs, physical education as well as compliance with physical activity guidelines. Its test-retest reliability and validity was tested and found to be comparable to other internationally published PA questionnaires for children and adolescents (test-retest reliability: ICC = 0.68) [47, 48].

We use the pooled dataset from wave 1 and wave 2 (total *n* = 11,337). We cannot use data from the baseline, because the mediator variables were not measured. Our data analyses include children aged between six and 17. The great majority of children and adolescents of this age attend school and potentially engage in organized and unorganized sports in their leisure time (afternoon, evenings, and weekends). Due to the panel design of the study, one respondent contributes one or two observations to the study. The selection by age leaves us with 8411 measurements from 7127 children and adolescents. Table 1 summarizes the distribution of all variables. Note that the number of cases is affected by missing values; the lowest number of valid cases is for migration background. All analyses will apply a listwise deletion of cases with missing information, resulting in *N* = 6100 for organized sports, *N* = 6044 for unorganized sports and *N* = 5977 when combining both (i.e. no missing values in the covariates as well as organized *and* unorganized sports).

**Table 1:** Summary of variables

Variable	N	%	Mean	Median	SD	Min.	Max.
**Organized sports**	8,259		124.23	90.00	141.14	0.00	1,250
**Unorganized sports**	8,169		58.93	0.00	114.85	0.00	1,440
**SES** (z-score)	8,126		0.00	-0.07	1.00	-2.80	1.98
- Low	1,641	20.19					
- Medium	4,861	59.82					
- High	1,624	19.99					
**Opportunities (z-score)**	7,864		0.00	0.03	1.00	-3.70	2.61
- No	3,491	44.39					
- Yes	4,373	55.61					
**Support (z-score)**	7,883		0.00	0.00	1.00	-2.84	1.70
- No	3187	40.43					
- Yes	4696	59.75					
**Gender**	8,411						
- Male	4,147	49.30					
- Female	4,264	50.70					
**Migration background**	7,213						
- No	6,223	86.27					
- Yes	990	13.73					
**Age**	8,411						
- 6–10	2,918	34.69					
- 11–13	2,367	28.14					
- 14–17	3,126	37.17					
**Cohort**	8,411						
- 1991–2000	3,498	41.59					
- 2001–2011	4,913	58.41					
**District size**	8,395						
- <20k	4,653	55.43					
- 20k+	3,742	44.57					

(Source: MoMo/KiGGS)

The explaining variables are parental SES, gender, and migration background; the mediators are opportunities and family and peer support. In our sample of analysis, the gender composition of the children is well balanced and around 15% of the participants have a migration background (i.e. they are first- or second-generation migrants). The study design allows us to identify both age and cohort effects. Age controls for a potential *age-specific pattern in PA*, while comparing cohorts aims to analyze *trends* over (historical) time. We group children into two birth cohorts: 1991–2000 and 2001–2011. While children born in the 1990s grew up before the digital revolution, the millennials (2001–2011) experienced a significant shift in using digital media [49]. Finally, district size roughly captures urban (20,000 and more inhabitants) versus rural (under 20,000 inhabitants) environments, which might as well influence PA. Thus, the control variables for our study are age, birth cohort, and district size.

### Measures: SES, opportunities, support, organized and unorganized sports

The variable measuring parental SES is a composite score with values from 3.0 to 21.0 combining the following components: educational qualification, occupational status, and net income of the parents. Each dimension is assigned one to seven points. For calculating the SES score, the highest values of the educational qualification and the occupational status of either mother or father and the net income of the household (mother and father) are summed up [50]. The use of a composite measure of SES, combing fathers’ and mothers’ status characteristics, seems to be adequate to capture joint parental resources even if the separate impacts of fathers’ and mothers’ own PA on their children’s PA and motoric development are not identical.^3^ The composite measure is z-standardized to have a mean of zero and a standard deviation of one, which makes the coefficients comparable in size. For the descriptive analysis, the SES score is categorized into low, medium, and high SES. For the categorization, the metric SES score is divided into five quintiles. The middle three quintiles are combined to form the medium SES, so that the 60 middle percent of children fall into that category. The lowest quintile becomes the low SES group (lowest 20%) and the highest quintile becomes the high SES group (highest 20%) [52]. According to this categorization, the majority of children and adolescents have a medium parental SES (59.82%) while groups of similar size have a low (20.19%) and a high SES (19.99%) (for more details see Fig. 6 in the Appendix).

To measure opportunities in residential areas and family and peer support, we built indices combining multiple answers in each dimension. The opportunity score contains ratings of the walkability of the residential area, playgrounds, sports club, gyms, and a self-evaluation of how pleasant it is to move about the residential area on foot or by bicycle. All items were answered on a four-point rating scale (e.g. very unpleasant, somewhat unpleasant, somewhat pleasant, very pleasant). Table 2 shows all items. The index was built by adding up the answers of all individual items and dividing them by the number of items resulting in values from one to four. A test of the opportunity index found moderate to good reliability and construct validity [53]. For the descriptive analysis, the variable *opportunities* is dichotomized by median split into *opportunities* (yes; values from 2.875 to 4) and *no opportunities* (no; values from 1.00 to under 2.875).

**Table 2:** Composition of the opportunities and support variables

Variable	Items
Opportunities	In the area I live in, shops and businesses can be reached on foot.
	From where I live, the bus and tram stops can be reached on foot.
	In the area I live in, there are sports facilities that are always accessible (e.g. soccer fields).
	In the area I live in, there are playgrounds.
	How safe are the public leisure time facilities in the area you live in (in terms of problems with crime)?
	For walking and riding a bicycle, the area I live in is … (“not very nice at all” to “very nice”).
	In the area I live in, there are sports clubs.
	In the area I live in, there are commercial sports providers (e.g. fitness clubs).
Support	Does your father regularly do sports?
	Does your mother regularly do sports?
	Is your father member of a sports club?
	Is your mother member of a sports club?
	Does at least one of your siblings^*^ regularly do sports?
	Is at least one of your siblings^*^ member of a sports club?
	Does at least one of your friends regularly do sports?
	Is at least one of your friends member of a sports club?

Source: adapted from Reimers et al., 2012 [52]

*Children without siblings get assigned the value 0.5

The support score measures if the respondent’s parents, siblings, and peers engage in sports and if they are members of a sports club with eight dichotomous questions, which are also shown in Table 2. For the index, all answers were added up, resulting in values of whole numbers from zero to eight. For the questions on siblings’ sports engagement, children without siblings were assigned the value 0.5. For the descriptive analysis, the variable *support* is dichotomized by median split into *support* (yes, values from 5 to 8) and *no support* (no; values from 0 to 4). As for SES, the index measures of opportunities and support are z-standardized in the multivariate models.

PA in sports is measured by the number of minutes a respondent reports engaging in organized and unorganized sports in a regular week. In addition, respondents were asked in which months they engage in a sport (e.g. Do you engage in this sport in January?). The number of months, in which the sport is done, was then divided by twelve and this factor was multiplied with the reported minutes of sports. This accounts for seasonality effects [54]. Organized sports is defined as sports done in a sports club, unorganized sports describes sports done in leisure time outside of a sports club (i.e. not including school sports).

**Fig. 2:**
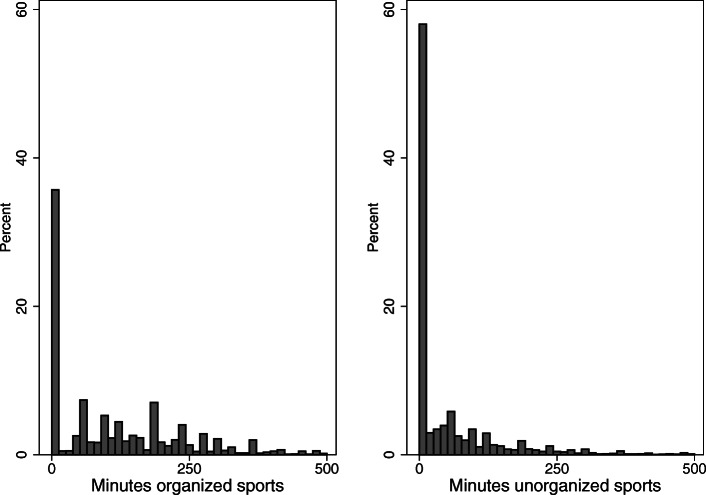
Distribution of the dependent variables. For a better overview, the number of minutes of PA in organized sports and unorganized sports are restricted to < 501 min: N(organized) = 8083, N(unorganized) = 8074 (source: MoMo/KiGGS)

Figure 2 displays the distributions of the number of minutes reported engaging in organized and unorganized PA per week. Both distributions are highly left-skewed and reveal an excess of zeros, i.e. a considerable proportion of children and adolescents report no activities in organized (34.73%) and unorganized sports (55.99%). Median values indicate that half of the respondents engage in more than 90 min per week of organized sports while half of the respondents do not engage in unorganized sports at all. Interestingly, both kinds of PA are uncorrelated (Pearson’s *r* = 0.001 *p* = 0.928). We account for the specific distributions using adequate statistical models (see below).

## Methods

To provide an overview of the differences in PA, the average number of minutes of PA in organized and unorganized sports are compared across groups. To test the statistical significance of these differences, Wilcoxon rank-sum tests [55] are conducted.^4^ To test the conceptualized relation between SES and opportunities/support, their correlation is estimated. For the multivariate regression analysis, we apply Cragg’s [56] double-hurdle model that considers the skewed distribution of the dependent variables (see Fig. 2). Doing *zero* minutes of organized and unorganized sports have high peaks in their distribution. These values represent “hurdles”, either through not being member of a sports club or through not doing any kind of sports outside of a sports club. Cragg’s [56] model allows this “piling up” of observations at a given point [57], being composed of two processes or tiers estimated by a probit equation and a linear equation. In the present analysis, the probit equation calculates the chance of Y > 0 (i.e. the chance that the number of minutes of PA in sports are higher than zero).^5^ To make the results of the double-hurdle models interpretable in terms of changes in actual minutes of PA, the average marginal effects (AME) are calculated.^6^ Since few children have been measured twice (due to the fact that they participated in a panel study), we estimated coefficients with robust standard errors [59, 60] to account for violating the assumption of independent and identically distributed standard errors. As already pointed out, when comparing the effect sizes of the continuous variables included in the models, we calculate z-scores by subtracting the mean from the raw value for all individual measurements and then divide the difference by the standard deviation of the raw values (z-standardization). The estimated coefficients of these z-standardized variables can be compared directly in a common metric. The estimated coefficients provide information about changes in the number of minutes per week spent in organized and unorganized PA if the independent variables change by one standard deviation.

## Results

In the first part of this section, the descriptive results of the number of weekly minutes spent by children and adolescents in PA in organized and unorganized sports are presented differentiated by parental SES and other variables included in the analysis. The second part of the section contains the multivariate analysis that allows us to identify the correlation of parental SES with PA while at the same time considering the mediators opportunities and support.

Table 3 depicts the average minutes of PA in organized and unorganized sports for different groups, and their statistical significance according to Wilcoxon rank-sum tests. Almost all group differences are statistically significant. Children and adolescents with a high SES spend much more time on PA in organized sports (155 min) than children with a low SES (99 min), whereas the difference in unorganized sports is very small (58 vs. 61 min). Children and adolescents with opportunities for PA in their residential areas are more physically active in organized (141 min) and unorganized (63 min) sports than children without opportunities (108 and 54 min). Children and adolescents whose families and peers support their PA are much more physically active in organized sports (153 min) than respondents without support (86 min). Again, this difference is smaller for unorganized sports (61 vs. 55 min) than for organized sports. Boys are more physically active in organized (146 min) and unorganized (66 min) sports than girls (106 and 52 min). Respondents without a migration background spend more time being physically active in organized (127 min) and unorganized (60 min) sports than respondents with a migration background (117 and 56 min).

**Table 3:** Average weekly minutes of PA in organized and unorganized sports

		Organized sports	Unorganized sports
		Ø Minutes	Ø Minutes
**SES**	Low	99.12***	61.79***
	Intermediate	124.94	58.59
	High	155.21***	58.54***
**Opportunities**	No	107.68	53.82
	Yes	140.84***	63.54***
**Support**	No	86.21	55.28
	Yes	152.89***	61.83***
**Gender**	Male	146.12	66.13
	Female	105.96***	52.37
**Mig. back.**	No mig. back.	127.33	59.76
	Mig. back.	116.97**	55.51
**Age**	6–10	104.02***	42.62***
	11–13	141.13***	62.62***
	14–17	137.73	74.80
**Cohort**	1991–2000	139.54	74.81
	2001–2011	115.90**	47.60***
**District size**	< 20 k	121.70	61.30
	20 k+	131.40**	56.49**
**N**		6100.00	6044.00

Significance tested with Wilcoxom rank-sum tests: **p < 0.05, ***p < 0.001 (The tests indicate whether the mean values of doing organized sports (column 1) and unorganized sports (column 2) significantly differ by categories of the group variables. Note that for variables with three categories three tests are performed – each with one category against the two others as a common reference group.); (source: MoMo/KiGGS)

In terms of differences by control variables of age and cohort, adolescents are more physically active in organized and unorganized sports than younger children. Compared to the other two age groups, members of the middle age group (11–13 years) spend the most time being physically active in organized sports (141 min), followed by the oldest age group (14–17 years, 130 min) and the youngest age group (6–10 years, 104 min). In contrast, the age group of respondents has a somewhat linear effect on PA in unorganized sports. Again, adolescents are more physically active than younger children (75 vs. 43 min). The cohort differences are rather clear: the younger birth cohort engages less in organized (116 min) and unorganized (48 min) sports than the older birth cohort (140 and 75 min). Finally, children and adolescents from more rural areas (< 20 k) are less physically active in organized sports (122 min) than children and adolescents from more urban areas (20 k+, 131 min), although they are more active in unorganized sports (61 vs. 56 min).

**Fig. 3:**
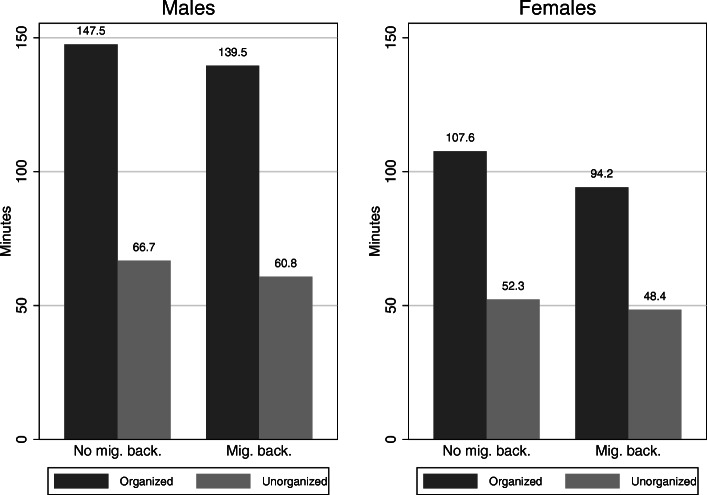
Average minutes of PA for males and females with and without a migration background (N(organized) = 6100; N(unorganized) = 6044; WRS-test: significant difference between females with and without migration background regarding organized sports) (source: MoMo/KiGGS)

Figure 3 additionally depicts the average weekly minutes of PA in organized and unorganized sports for males and females with and without a migration background. Boys without migration background engage more in organized sports (5.47% of boys’ total minutes), whereas boys with migration background engage slightly more in unorganized sports (0.15% of boys’ total minutes). Girls with a migration background are a little less physically active in both types of sports than their counterparts without a migration background (12.65% of girls’ total minutes in organized sports and 7.45% in unorganized sports). Only the difference for girls regarding organized sports is significant (WRS-test, *p* < 0.05). Comparing the differences between boys and girls, girls show a higher difference between migration background and no migration background.

Before we start the multivariate analysis, we report the correlations of the parental SES and the mediators (z-standardized variables, *N* = 5977) Parental SES and opportunities have a small positive correlation (*r* = 0.13, *p* < 0.001); the correlation of parental SES and support is also positive and higher (*r* = 0.30, *p* < 0.001). The mediators also correlate positively with each other (*r* = 0.17, *p* < 0.001).

Table 4 shows the AME (i.e. how much, on average, do the minutes for organized/unorganized sports change if continuous variables increase by one standard deviation resp. if dummy variables are compared against the reference group) estimated in the double-hurdle models. The first model for organized sports shows that children one standard deviation above the mean SES spend 21 min more being physically active in organized sports per week. Adding the mediators of opportunities and support (Model 2) decreases the size of the effect of parental SES to eight minutes. Adding the variables of gender and migration background (Model 3) barely affects the SES-effect size. Adding the controls of age, cohort, and district size changes the SES effect size to ten minutes in the final model. Comparing the effect sizes of ten and 21 min from Models 4 and 1, we learn that including mediators and control variables explains about half of the total parental SES effect size.

**Table 4:** AME of Cragg’s (1971) double-hurdle models. Models 3 and 4 include an interaction effect between gender and migration background

	Organized sports				Unorganized sports			
	(1)	(2)	(3)	(4)	(1)	(2)	(3)	(4)
SES (z-score)	21.43^***^	8.00^***^	7.68^***^	9.90^***^	−0.46	−2.42	− 2.48	0.15
	(1.87)	(1.88)	(1.85)	(1.86)	(1.44)	(1.51)	(1.51)	(1.52)
Opportunities (z-score)		13.78^***^	14.14^***^	12.17^***^		5.64^***^	5.82^***^	4.67^**^
		(1.83)	(1.82)	(1.82)		(1.44)	(1.44)	(1.45)
Support (z-score)		37.39^***^	37.09^***^	37.75^***^		3.96^**^	3.70^*^	3.81^**^
		(1.78)	(1.75)	(1.72)		(1.49)	(1.48)	(1.47)
Gender (female)			−45.53^***^	−45.64^***^			−12.34^***^	−12.62^***^
			(3.42)	(3.34)			(2.79)	(2.76)
Migration background			−7.84	−5.55			−6.09	−3.54
			(5.12)	(5.10)			(3.92)	(4.00)
Age (6–10)								
- 11-13				30.58^***^				8.92^*^
				(4.29)				(3.51)
- 14-17				24.46^***^				18.16^***^
				(4.86)				(3.93)
Cohort (1991–2000)								
- 2001-2011				−17.30^***^				−17.83^***^
				(4.29)				(3.48)
District size (20 k+)				5.48				−4.92
				(3.60)				(2.85)
N	6100.00	6100.00	6100.00	6100.00	6044.00	6044.00	6044.00	6044.00

*p < 0.10, **p < 0.05, ***p < 0.001 *SE in parentheses; (Source: MoMo/KiGGS)

The full model (Model 4) also reveals that scoring one standard deviation above the mean of the opportunities scale is accompanied with 12 more minutes being physically active in organized sports – in comparison to someone with average opportunities in the residential area. Having more family and peer support (again one standard deviation above the mean of the scale) increases the average minutes of PA in organized sports by 38 min. Thus, family and peer support have a much larger leverage than just having better opportunities. Taken together, these results are in line with *H1* for the impact of parental SES, *H2* for the relevance of opportunities, and *H3* for the relevance of support regarding organized sports.

**Fig. 4:**
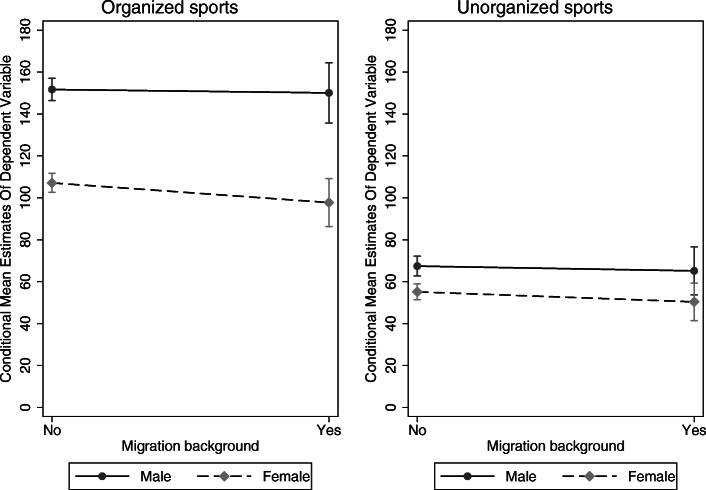
Predictive margins of gender and migration background on organized and unorganized sports (N(organized) = 6100; N(unorganized) = 6044; (source: MoMo/KiGGS)

On average, girls spend 46 min less on organized sports than boys in the final model. Children with a migration background spend about six minutes less on organized sports, but the difference is not significant (Model 4). Thus, we find empirical evidence for the gender differences proposed in *H4*, but no support for the differences regarding migration background proposed in *H5*. Models 3 and 4 additionally estimate interaction effects between gender and migration background, which cannot be transformed into AME. However, for illustration, Fig. 4 shows the predictive margins of gender and migration background on organized and unorganized sports. Migration background shows a small negative effect size, which is larger for girls than for boys, regarding organized and unorganized sports. Thus, there is only little evidence to support the gender-specific effect proposed in *H6*.

Regarding organized sports, children aged between 11 and 13 years are 31 min more physically active than children in the reference group (aged 6–10 years), and children aged 14–17 years are 24 min more physically active than children aged 6–10 years. Children from the younger birth cohort (2001–2011) spend 17 min less being physically active in organized sports than children from the older birth cohort (1991–2000). We find no effect of district size on organized sports.

In all models on unorganized sports, parental SES shows no significant effect at all, which contradicts *H1* in the domain of unorganized sports. Children and adolescents with an opportunities score one standard deviation above the mean are physically active in unorganized sports for 5 min more than children with an opportunity score at the mean. Children and adolescents with more family and peer support are physically active in unorganized sports for 4 min more compared to the mean value of the support scale (Model 4). These results underpin *H2* and *H3* but with a much lower slope compared to the domain of organized sports. Girls are 13 min less physically active in unorganized sports than boys. This finding supports *H4* in the field of unorganized sports. No effect of migration background on unorganized sports was found. Figure 4 shows a very small effect, which is stronger for girls than for boys. *H5* and *H6* are therefore rejected regarding unorganized sports. Children in the middle age group (11–13 years) are 9 min more physically active (barely significant), and children in the oldest age group (14–17 years) are 18 min more physically active than children in the youngest age group (6–10 years). Children in the younger birth cohort (2001–2011) spend 18 min less being physically active in unorganized sports than children in the older birth cohort (1991–2000).

**Fig. 5:**
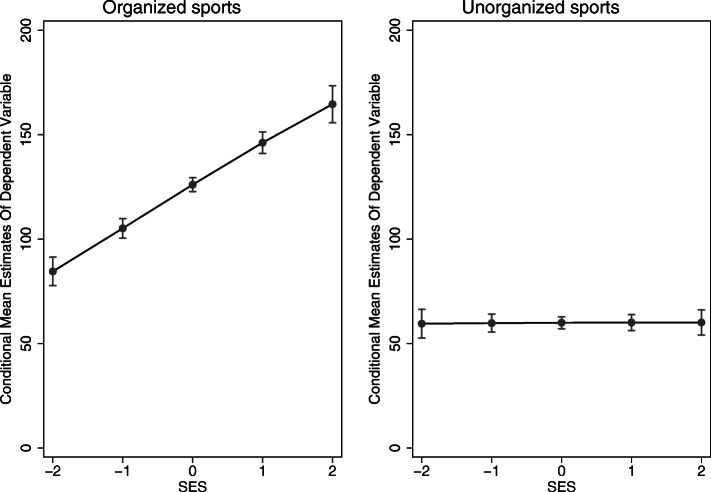
Predictive margins of SES on organized and unorganized sports (N(organized) = 6100; N(unorganized) = 6044) (source: MoMo/KiGGS)

**Fig. 6:**
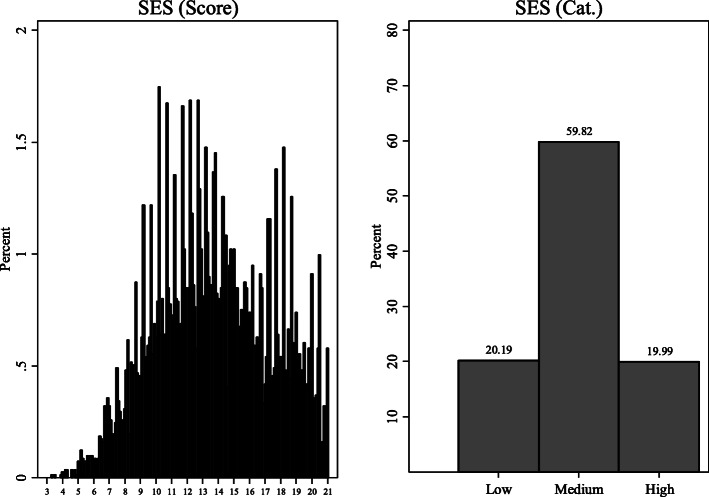
Distribution of the independent variable SES (score and categorized; *N* = 8126) (source: MoMo/KiGGS)

For comparison, Fig. 5 shows the predictive margins of SES on organized and unorganized sports for the full models (Model 4). There is a large positive slope for organized sports, whereas we see no effect of SES on unorganized sports. In other words, children and adolescents with a standardized SES of − 2 are physically active in organized (unorganized) sports for about 85 [61] minutes per week, whereas children with a standardized SES of 2 are physically active in organized sports for about 165 [61] minutes per week.

We further investigated interaction effects between SES and all other covariates to check, if the SES effect differs between the categories of the covariates, e.g. between male and female children. We find no significant interaction effects, i.e. the SES effect does not differ between the categories of the covariates in our analysis. The results are available from the authors on request.

## Discussion and conclusion

Our study contributes to the discussion about children’s parental background and its impact on health related PA – by a differentiated view on organized and unorganized sports and a mediator analysis with opportunity and support structures. In line with the result by Schmidt et al. [21] and Andersen and Bakken [17], our results clearly indicate that low parental SES correlates with more difficult access for children and adolescents to participation in organized sports. We also demonstrate that SES only marginally correlates with unorganized sports. The coefficient of parental SES on organized sports most probably relates to the resources needed to participate in sports clubs, including fees and equipment. The mediator analysis with opportunity and support scores revealed that parental SES has a smaller but independent effect size on PA.

These findings point to possible policy measures. In principle, a higher inclusion of children and adolescents with low parental SES seems to strengthen health-related outcomes. One potential measure might be reduced fees and sponsored equipment for children and adolescents with a low parental SES. Lower membership fees might potentially help to integrate children with low parental SES into sports clubs and thereby make organized sports more accessible to all social classes.

Regarding targeting measures by gender and migration background, in general, girls seem to be less physically active in organized and unorganized sports than boys – in particular, girls with a migration background engage less in organized sports. Again, this finding could legitimate measures to strengthen support for girls’ PA, particularly in families with a migration background. The gender issue might be even more relevant to adolescents than to young children. When controlling for SES, the effect of migration background is no longer significant. Since migration background oftentimes correlates with low SES, this result indicates that the effect of migration background found in previous studies [38], instead is an SES effect. Future research needs to examine the relation between migration background, gender, age, and (organized) sports more closely. For future research, it would be also desirable to have more precise information on the migration background because we can expect major differences among migrants from different countries.

Finally, the comparison of birth cohorts shows another interesting result that deserves some reflection. The younger birth cohort (2001–2011) is less physically active in organized and unorganized sports than the older birth cohort (1991–2000). One reason might be the negative correlation between the increasing use of digital media and PA [61, 62]. Therefore, younger cohorts might use more digital media, which increases their sedentary time and decreases their PA. Again, family support for PA is a crucial mediator when confronted with the overall trend of less organized and unorganized sports. A further contextual feature that is not covered in this study might be the increased dissemination of all-day schooling in Germany. However, research results on the PA of children in all-day versus half-day schooling are mixed [63]. Further research comparing the PA of current birth cohorts is necessary to ascertain whether sports participation is decreasing in younger cohorts and, if so, the reasons for the decrease.

Summarizing the results, it can be concluded that children and adolescents who have a higher parental SES, who live in a residential area with more opportunities for PA, and whose families and peers support PA spend more time being physically active in *organized* sports than children and adolescents without these assets. Furthermore, males and adolescents are more physically active in organized sports than females and younger children. We found less pronounced effects for unorganized sports. Probands who live in a residential area with a lot of opportunities for PA, probands whose families and peers support their PA, and males and older probands are more physically active in unorganized sports than their counterparts. Overall, this study shows that differences in parental SES mainly affect organized sports and have little to no influence on unorganized sports. Therefore, sports clubs need to work towards integrating children from families with a low social status, as well as girls and children with a migration background.

When discussing the results and conclusions of the present study, its limitations and strengths have to be considered as well. An obvious limitation of the study is the self-reported measurement of PA, especially by young children. All interviews with children in the age group of 6–10 years were attended by a parent of the child or another responsible adult to help the children give accurate answers [47]. Self-report methods are potentially imprecise and biased by social desirability, but they are widely used in large sample studies. Because device-based measures of PA (e.g. accelerometers) are also subject to some methodological problems like short measuring intervals [64]. Therefore, we decided to rely on the self-reported PA estimate for this study. A major strength of our measurement of PA in minutes per week is its easy and precise interpretation by changes in actual minutes.

One could also question whether our separate analysis of PA in organized and unorganized sports is adequate given our focus on the relevance of PA in general. For the positive outcomes of PA, it does not matter whether PA was organized; however, both concepts do not correlate at all (*r* = 0.001, *p* = 0.982). Obviously, time availability is a massive restriction on both organized and unorganized sports, but, more importantly, for analytical reasons, the distinction between organized and unorganized sports is very informative. Accordingly, we identified marked differences between the two settings (for example, parental SES being relevant to organized sports only). Finally, we consider it to be a strength of this study that we used hurdle models to differentiate between overcoming hurdles in the way of being physically active in sports *at all* (Y > 0) and the *amount* of PA in sports (see the results of both tiers of estimations in Tables 5 and 6 in the Appendix).

Another limitation to consider is the construction of the support index. Adding up all questions on the respondents’ parents’, siblings’, and peers’ sports and sports club engagement assumes first that parents’, siblings’, and peers’ sports engagement is equally important for the sports engagement of the children. Second, it assumes that sports engagement and sports *club* engagement are equally important for children’s sports and sports *club* engagement respectively. To further disentangle the relationship between parents’, siblings’, and peers’ sports and sports club engagement and children’s PA in organized and unorganized sports additional research is needed.

Furthermore, we must consider the possible endogeneity between both mediators opportunities and family and peer support with children’s PA. For example, the sports engagement of the peers might not only lead to higher PA of a respondent, but the higher PA of a respondent might also lead to higher sports engagement of the peers. PA opportunities in the neighborhood might not only result in higher PA, but higher interest in PA might also result in better knowledge of opportunities for PA in the neighborhood. We cannot solve this problem completely in our analysis. However, we reiterate that we do not aim at drawing causal conclusion from our analysis, but at a description of correlations. We can confirm the correlation between the mediators and children’s sports engagement, and we assume that the mechanisms proposed in this study (the mediators influencing PA) at least partially explain this correlation.

What becomes crystal-clear after reflecting strengths and limitations of the study at hand: further research would enormously profit from representative longitudinal panel data with more frequent points of measurement (in order to better identify effects of changes in the independent variables), ideally with a combination of self-reported and device-based measures and with the chance to link individual data to context information on schools and neighborhoods.

## Data Availability

The data set cannot be made publicly available because informed consent from study participants did not cover public deposition of data. However, the minimal data set underlying the findings is archived at the Institute of Sports and Sports Science of the Karlsruhe Institute of Technology (KIT) and can be accessed by interested researchers on site. On-site access to the minimal data set should be submitted to Prof. Dr. Alexander Woll, Karlsruhe Institute of Technology, Engler-Bunte-Ring 15, 76131 Karlsruhe, Germany (alexander.woll@kit.edu).

## Appendix

**Table 5:** Results of the double-hurdle models for organized sports

Organized sports	(1)		(2)		(3)		(4)	
	Linear	Probit	Linear	Probit	Linear	Probit	Linear	Probit
**SES** (z-score)	0.03^**^	0.25^***^	0.01	0.12^***^	0.01	0.12^***^	0.03^*^	0.11^***^
	(0.01)	(0.02)	(0.01)	(0.02)	(0.01)	(0.02)	(0.01)	(0.02)
**Opportunities** (z-score)			0.09^***^	0.05^**^	0.09^***^	0.05^**^	0.06^***^	0.07^***^
			(0.01)	(0.02)	(0.01)	(0.02)	(0.01)	(0.02)
**Support** (z-score)			0.07^***^	0.49^***^	0.07^***^	0.50^***^	0.07^***^	0.49^***^
			(0.01)	(0.02)	(0.01)	(0.02)	(0.01)	(0.02)
**Gender** (female)					− 0.23^***^	−0.26^***^	− 0.23^***^	−0.25^***^
					(0.02)	(0.04)	(0.02)	(0.04)
**Mig. back.** (yes)					−0.05	0.04	−0.03	0.04
					(0.05)	(0.07)	(0.04)	(0.08)
**Gender#mig. Back.**					−0.01	−0.13	−0.02	−0.12
					(0.07)	(0.10)	(0.07)	(0.10)
**Age**								
- 11-13							0.26^***^	−0.04
							(0.03)	(0.05)
- 14-17							0.33^***^	−0.28^***^
							(0.03)	(0.05)
**Cohort** (2001–2011)							−0.14^***^	0.02
							(0.03)	(0.04)
**District size** (20 k+)							0.06^**^	−0.05
							(0.02)	(0.04)
Constant	5.01^***^	0.44^***^	4.99^***^	0.48^***^	5.10^***^	0.62^***^	4.98^***^	0.74^***^
	(0.01)	(0.02)	(0.01)	(0.02)	(0.02)	(0.03)	(0.03)	(0.06)
Lnsigma^a^ Constant		−0.34^***^		−0.36^***^		−0.37^***^		−0.41^***^
		(0.01)		(0.01)		(0.01)		(0.01)
N		6100.00		6100.00		6100.00		6100.00

**p* < 0.10, ***p* < 0.05, ****p* < 0.001 *SE in parentheses; (Source: MoMo/KiGGS)

^a^ Information about standard deviation of error term

**Table 6:** Results of the double-hurdle models for unorganized sports

Unorganized sport	(1)		(2)		(3)		(4)	
	Linear	Probit	Linear	Probit	Linear	Probit	Linear	Probit
**SES** (z-score)	−0.05^**^	0.05^**^	−0.05^**^	0.01	−0.05^**^	0.01	−0.04	0.05^**^
	(0.02)	(0.02)	(0.02)	(0.02)	(0.02)	(0.02)	(0.02)	(0.02)
**Opportunities** (z-score)			0.06^**^	0.04^*^	0.06^**^	0.04^**^	0.05^*^	0.04^*^
			(0.02)	(0.02)	(0.02)	(0.02)	(0.02)	(0.02)
**Support** (z-score)			−0.01	0.09^***^	−0.01	0.09^***^	−0.01	0.09^***^
			(0.02)	(0.02)	(0.02)	(0.02)	(0.02)	(0.02)
**Gender** (female)					−0.20^***^	−0.00	−0.19^***^	− 0.01
					(0.04)	(0.03)	(0.04)	(0.04)
**Mig. back.**					−0.02	−0.07	−0.01	− 0.03
					(0.08)	(0.07)	(0.08)	(0.07)
**Gender#mig. Back.**					−0.08	0.03	−0.08	0.02
					(0.11)	(0.09)	(0.11)	(0.10)
**Age**								
- 11-13							0.17^***^	−0.01
							(0.05)	(0.04)
- 14-17							0.15^**^	0.17^***^
							(0.05)	(0.05)
**Cohort** (2001–2011)							−0.08	−0.24^***^
							(0.04)	(0.04)
**District size** (20 k+)							0.01	−0.11^**^
							(0.04)	(0.03)
Constant	4.48^***^	−0.13^***^	4.48^***^	−0.13^***^	4.58^***^	−0.12^***^	4.51^***^	0.01
	(0.02)	(0.02)	(0.02)	(0.02)	(0.03)	(0.02)	(0.05)	(0.05)
Lnsigma Constant		−0.07^***^		−0.07^***^		−0.08^***^		−0.09^***^
		(0.01)		(0.01)		(0.01)		(0.01)
N		6044.00		6044.00		6044.00		6044.00

**p* < 0.10, ***p* < 0.05, ****p* < 0.001 *SE in parentheses; (source: MoMo/KiGGS)

## Abbreviations

AME: Average marginal effect(s)
IfSS: Institute of Sports and Sports Science
KiGGS: Health Interview and Examination Survey for Children and Adolescents
KIT: Karlsruhe Institute of Technology
MoMo: Motorik-Modul-Study
PA: Physical activity
RKI: Robert Koch Institute
SES: Socioeconomic status
WHO: Word Health Organization
WRS-test: Wilcoxon-ranksum-test

## Footnotes

1 Since we include respondents from the age of six to the age of 17, the term “children and adolescents” is appropriate. However, to increase readability, we mainly use the term “children” from now on.

2 Studies in the tradition of Pierre Bourdieu [24] analyze whether sports activities (either performed or preferred watching) depend on social class background [25]. Doing sports reflects actors’ strategies of social distinction. We do not refer to this branch of research because we are not able to identify Bourdieu’s concepts such as *habitus* in the available data on children and adolescents.

3 Mothers’ PA level seems to be relevant for daughters while fathers’ PA level tends to influence the PA of sons [51].

4 In case of more than two categories, the difference between one category and all other categories is tested, resulting in separate tests for each category.

5 We therefore assume that the zero-observations are “censored zero after”, i.e. the respondents doing zero minutes of sports is determined by current circumstances, as opposed to the respondents being “zero types”, which under no circumstances would do more than zero minutes of sports [58].

6 The results of the double-hurdle models are presented in Table 5 and Table 6 in the Appendix.
